# Myopathic Symptoms and Exercise Tolerance in Adolescent Patients With Long‐Chain Fatty Acid Oxidation Disorders

**DOI:** 10.1002/jimd.70070

**Published:** 2025-07-29

**Authors:** Marit Schwantje, Marco van Brussel, Tim Takken, Monique G. M. de Sain‐van der Velden, Mirjam Langeveld, Gepke Visser, Sabine A. Fuchs

**Affiliations:** ^1^ Department of Metabolic Diseases Wilhelmina Children's Hospital, University Medical Center Utrecht Utrecht the Netherlands; ^2^ Child Development and Exercise Center Wilhelmina Children's Hospital, University Medical Center Utrecht Utrecht the Netherlands; ^3^ Department of Medical Genetics and Wilhelmina Children's Hospital University Medical Center Utrecht Utrecht the Netherlands; ^4^ Department of Endocrinology and Metabolism Amsterdam UMC, University of Amsterdam, Amsterdam Gastroenterology Endocrinology Metabolism (AGEM) Research Institute Amsterdam the Netherlands; ^5^ Emma Children's Hospital, Department of Pediatrics, Division of Metabolic Diseases, Amsterdam Gastroenterology Endocrinology and Metabolism Amsterdam UMC, Location University of Amsterdam Amsterdam the Netherlands

**Keywords:** cardiopulmonary exercise testing, long‐chain fatty acid oxidation disorders (lcFAOD), newborn screening, prolonged exercise testing, rhabdomyolysis, very long‐chain acyl‐CoA dehydrogenase deficiency

## Abstract

Long‐chain fatty acid oxidation disorders are characterized by rhabdomyolysis, often provoked by physical exercise. For the newborn screening (NBS) cohort, it remains uncertain to what extent they will develop the myopathic phenotype. This study assesses physiological responses to exercise, muscle symptoms, and activity levels in 14 adolescent lcFAOD patients (VLCADD (*n* = 8), LCHADD (*n* = 4), CPT2D (*n* = 1) and LCKATD (*n* = 1); ages 9.9–17.8 years). Analyses of incremental and prolonged cardiopulmonary exercise tests, a symptom‐based questionnaire, and the Short Questionnaire to Assess Health‐enhancing physical activity were performed. The results revealed a decreased ventilatory anaerobic threshold compared to control data (z‐score − 0.5 (0.8) [median (interquartile range (IQR))], *p* = 0.001) and, on average, a decreased relative peak oxygen uptake (z‐score − 1.3 (2.8), *p* = 0.005) and relative peak work rate (z‐score − 0.7 (1.3), *p* = 0.03). There were no adverse events during and following prolonged exercise under well‐fed circumstances (based on symptoms and post‐exercise creatine kinase). The symptom‐based questionnaire revealed that the presence of provoking factors (e.g., infection, inadequate intake) increased the risk of rhabdomyolysis during/after exercise. Screening (*n* = 11) and symptomatically (*n* = 3) diagnosed patients showed normal levels of physical activity (medians: 3.5 h per week) compared to their healthy peers (3.2 h), despite debilitating muscle pain in 46% of the by screening and all of the symptomatically diagnosed patients. In conclusion, patients with seemingly normal exercise patterns reported debilitating muscle symptoms and rhabdomyolysis, especially when additional provoking factors were present. Exercise tests may provide a valuable tool to monitor and guide exercise potential in these new NBS cohorts.

## 1 Introduction

Long‐chain fatty acid oxidation disorders (lcFAOD) represent a group of inherited metabolic disorders caused by a deficiency of one of the enzymes involved in long‐chain fatty acid oxidation [1, 2]. During infancy, these disorders mainly cause hypoglycemia and cardiomyopathy [1, 2]. Starting in adolescence, myopathy, characterized by recurrent episodes of rhabdomyolysis, can occur, often provoked by physical exercise [1, 3].

Newborn screening (NBS) for lcFAOD was introduced in the Dutch NBS panel in 2007 [3]. As the oldest patients diagnosed by NBS have just entered adolescence, it is still unknown whether this NBS cohort, predominantly diagnosed pre‐symptomatically and treated with diet as early as possible, is at risk to develop myopathic symptoms [3]. Managing the potential risks of physical exercise presents a difficult balance between social and long‐term health benefits versus acute risks of exacerbations. A ‘want to be on the safe side policy’ may induce a pattern of hypo‐activity and thereby precipitate risks for deconditioning and subsequent health problems (e.g., cardiovascular diseases, impaired musculoskeletal and mental health) [4, 5].

The consensus‐based guidelines for very long‐chain acyl‐CoA dehydrogenase deficiency (VLCADD, OMIM #201475), the most common lcFAOD, encourage regular physical activity to promote the accretion and retention of lean body mass [6]. However, individual exercise recommendations remain difficult to establish as exercise should be adjusted according to the individual patient's exercise tolerance, dietary regimen, and clinical symptoms. Unfortunately, there are no studies to guide exercise prescription for patients with lcFAOD [7], nor any known patient characteristic, biomarker, or response to exercise to predict exercise capacity. A deeper understanding of myopathic symptoms in patients with lcFAOD and establishment of their individual limits of safe exercise and exercise intensity are crucial to provide personalized exercise recommendations and minimize the risk of rhabdomyolysis resulting from physical activity.

Exercise testing can give insight into individual exercise tolerance and exercise‐related risks. Gas analysis during exercise can assess utilization of the different energy systems and a variety of physiological responses to exercise. Patients with lcFAOD are anticipated to rely more heavily on non‐fat energy sources, such as carbohydrates. This shift in energy utilization can be monitored during exercise tests. For lcFAOD, there is no known optimal standardized exercise test to determine exercise capacity and exercise‐related risks. Incremental cardiopulmonary exercise testing (CPET) is considered the golden standard to determine exercise capacity and provides well‐validated physiological information regarding overall health that might be utilized for diagnostic, prognostic, and evaluative purposes [8]. In contrast to the short and intensive CPET, prolonged endurance exercise testing (PXT) may be most relevant for lcFAOD [9, 10, 11, 12, 13], as it allows testing of the fat oxidation‐dependent energy production.

To increase understanding of the disease manifestations and patients' exercise capacity, this study reports the muscle symptoms, activity levels, and physiological responses to exercise in 14 adolescent patients with lcFAOD.

## 2 Methods

Patients were identified via the Dutch expertise center for lcFAOD at the Wilhelmina Children's Hospital (WKZ), University Medical Center Utrecht (UMCU), the Netherlands. Included patients were detected by NBS (*n* = 10), by sibling screening (*n* = 1), or after clinical presentation with exercise‐ or illness‐induced rhabdomyolysis (*n* = 3). These three patients were either missed by NBS (long‐chain 3‐hydroxyacyl‐CoA dehydrogenase deficiency (LCHADD), diagnosed at 9 months of age, OMIM #609016), had an lcFAOD not included in the Dutch NBS panel (carnitine palmitoyltransferase type 2 deficiency (CPT2D), diagnosed at the age of 8 years old, OMIM #255110) or were born before the introduction of the lcFAOD in the Dutch NBS panel (long‐chain 3‐ketoacyl‐CoA thiolase deficiency (LCKATD), diagnosed at the age of 17 years, no OMIM entry). All diagnoses were confirmed by biochemical and genetic analysis. Upon approval by the medical ethics committee of the UMCU (MEC20‐335C), patients performed a cardiopulmonary exercise test (CPET) and a combined intermittent‐ and prolonged‐endurance exercise test as part of their clinical follow‐up. Informed consent was obtained from all patients/parents.

### 2.1 Daily Physical Activities and Myopathic Symptoms

Before exercise testing, patients and parents were asked standardized questions regarding physical activities, (myopathic) symptoms, and dietary measures. To allow for comparison of daily life physical activities with the Dutch pediatric population, the Short Questionnaire to Assess Health‐enhancing physical activity (SQUASH) was used [14, 15]. Discrimination was made between mild and debilitating muscle pain (defined as muscle pain leading to school absence, hospital admission, staying in bed/on the couch, or not being able to walk or bike properly). Following the exercise tests, patients were contacted by phone every three to six months to assess exercise‐induced myopathic symptoms and physical activity levels.

To increase insight in the occurrence of exercise‐induced muscle pain in non‐lcFAOD children, we designed and performed a questionnaire assessment of the frequency and intensity of exercise‐induced muscle soreness (Supporting Information) in children with medium‐chain acyl‐CoA dehydrogenase deficiency (MCADD) or phenylketonuria (PKU), diagnosed by NBS and identified via the outpatient clinics of the WKZ, and healthy children (siblings of included patients). In patients with MCADD or PKU, (preventive) dietary measures are recommended after diagnosis by NBS, like in patients with lcFAOD, but these patients are not typically at risk for exercise‐induced rhabdomyolysis. Chronic complaints of fatigue, muscle pain, and reduced exercise capacity have been reported for pre‐NBS patients with MCADD, but not for PKU [16, 17, 18].

### 2.2 Cardiopulmonary Exercise Test (CPET)

All patients performed an incremental CPET on the bicycle ergometer according to the Godfrey protocol [19] until the patient stopped due to volitional exhaustion. During the test, the subjects breathed through a facemask (Hans Rudolph Inc., Kansas City, Missouri, USA) connected to a calibrated respiratory gas analysis system (Ergostik, Geratherm; Accuramed, Lummen, Belgium) which measured and/or calculated breath‐by‐breath minute ventilation (VE), oxygen uptake (VO_2_), carbon dioxide production (VCO_2_, ml/kg/min), and respiratory exchange ratio (RER; calculated by VCO_2_·VO_2_^−1^) using conventional equations. Heart rate (HR, beats per minute (bpm)), rhythm, and conduction were measured continuously by a 10‐lead electrocardiogram. Peak values of RER, HR, VO_2_, and work rate (WR) were calculated from breath‐by‐breath data averaged over nine breaths, thereby representing the mean of the last 30 s. The ventilatory anaerobic threshold (VAT) was calculated using the equivalents method, using the VE/VO_2_ to detect the first increase in VE/VO_2_. Peak values of oxygen uptake (VO_2peak_) and work rate (WR_peak_) were normalized for body mass to diminish the influence of body size as well as to adjust for differences in muscle mass. Absolute and relative (normalized for body mass) VAT, VO_2peak_, WR_peak_, and HR_peak_ were reported as median z‐scores with interquartile range (IQR). Z‐scores were calculated based on previously reported normal values, corrected for age and gender [20], using the formula: z‐score = (χ—μ)/σ (χ = reported value; μ = mean of the normal population, σ = SD of normal the normal population). Efforts were considered maximal if HR_peak_ was > 180 bpm or the RER_peak_ was > 1.0 [20]. On the days of the exercise tests, patients were instructed to take their regular pre‐exercise dietary measures.

### 2.3 Combined Intermittent (IXT)‐ and Prolonged Endurance Exercise Test (PXT)

**FIGURE 1:**
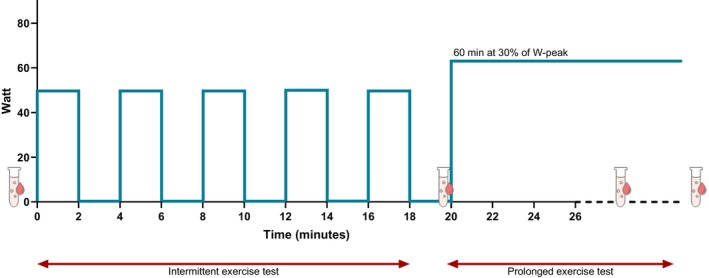
The combined intermittent (IXT) and prolonged endurance exercise test (PXT) protocol. The IXT consisted of five bouts of constant‐load exercise (2 min at 50 W per bout, 60–80 rotations per minute (rpm)) followed by periods of unloaded cycling (2 min at 0 W per bout, 60–80 rpm). The PXT consisted of 60 min of cycling at a constant workload at 30% of individual WR_peak_ attained during the CPET. Blood samples were collected pre‐exercise (time point (T) = 0 min), after the last bout of the IXT (T = 20), after 30 min of prolonged endurance exercise (T = 50), post‐exercise (T = 80) and after 30 min of recovery (T = 110).

A combined intermittent‐ and prolonged endurance exercise test was performed one week after CPET. The intermittent exercise test protocol (IXT) consisted of five bouts of constant‐load exercise followed by periods of unloaded cycling (Figure 1) and was performed as a potential longitudinal follow‐up measurement throughout adulthood. The prolonged endurance exercise test (PXT), which started directly after the last bout of unloaded cycling of the IXT, consisted of 60 min of cycling at a constant work rate at 30% of WR_peak_ attained during the CPET (Figure 1). With this, we aimed for low‐intensity exercise to stimulate fat oxidation [21].

During the PXT, RER, HR, VO_2_, and VCO_2_ were monitored using the same settings as reported for CPET. Using breath‐by‐breath data, the highest (RER_peak_) and lowest (RER_nadir_) RER values during unloaded and loaded cycling were determined for IXT. For PXT, mean RER and whole‐body rates of carbohydrate and fat oxidation (in g min^−1^) were calculated from the rates of CO_2_ production and O_2_ uptake, conceding that VO_2_ and VCO_2_ accurately reflect tissue O_2_ uptake and CO_2_ production. The following equations were applied [22]: Carbohydrate oxidation = 4.585 × VCO_2_ (l·min^−1^)−3.226 × VO_2_ (l min^−1^); and: fat oxidation = 1.695 × VO_2_ (l min^−1^)−1.701xVCO_2_ (l min^−1^). In these equations, energy provided from carbohydrate and fat oxidation was calculated from their energy potentials (3.87 and 9.75 kcal g^−1^, respectively) [22].

### 2.4 Blood Analyses

For CPET, blood samples were collected pre‐exercise and at two minutes post‐exercise and comprised lactate, creatine kinase (CK), N‐terminal pro–B‐type natriuretic peptide (NT‐proBNP) and troponin measurements. CK and cardiac enzymes were measured to evaluate the skeletal muscles and the heart, the predominantly affected organs in lcFAOD. For IXT and PXT, blood samples were taken as indicated in Figure 1. Patients were instructed not to eat or drink sugar‐containing drinks. Glucose, lactate, ammonia, CK, cardiac enzymes, ketone bodies, free fatty acids (FFA), and acylcarnitines were measured in all blood samples. Patients went to a local hospital the following day (~24 h after testing) for post‐testing measurement of CK, cardiac enzymes, and acylcarnitines.

### 2.5 Comparison to Other Metabolic Diseases

For validation of substrate oxidation results, patients with lcFAOD were compared to three patients with other metabolic diseases, who performed the exercise tests as part of their clinical follow‐up. These included two patients with glycogen storage disease (GSD) (type III and IXd, 16 and 14 years old, respectively), and one with impaired mitochondrial oxidation (acyl‐CoA dehydrogenase 9 deficiency, (ACAD9D), 18 years old).

### 2.6 Statistical Analyses

Statistical analysis was performed using SPSS (version 29.0.0.0, SPSS IBM, New York). Depending on the distribution, student *t*‐tests or Mann–Whitney U‐tests were used to compare between groups. To compare patients' data with the normal population, mean values of the population reported by the CBS [23, 24] were assumed to be similar to the hypothetical median values in large sample sizes. One‐sample Wilcoxon Signed Rank Tests were used to compare CPET results of patients (in z‐scores) to reported control data (z‐score = 0) [20]. Potential correlations were analyzed using Spearman rank correlations. *p*‐values below 0.05 were considered significant.

## 3 Results

Fourteen patients with a median age of 12.7 years (range: 9.9–17.8) and various lcFAOD diagnoses (VLCADD (*n* = 8), LCHADD (*n* = 4), CPT2D (*n* = 1) and LCKATD (*n* = 1)) were included. The patients' genetic, biochemical, and clinical characteristics are presented in Table 1.

**TABLE 1:** Genetic and biochemical characteristics of included patients with lcFAOD.

VLCADD	Gene	Nucleotide change	Protein change	lcFAO flux	Enzyme activity VLCAD^b^		Diagnosis
PID1	*VLCAD*	c.848 T>C; c.848 T>C	p.Val283Ala; p.Val283Ala	77.5%	18%		NBS
PID2	*VLCAD*	c.1322G>A; c.1468G>C	p.Gly441Asp; p.Ala490Pro	45.0%	7%		NBS
PID3	*VLCAD*	c.848 T>C; c.848 T>C	p.Val283Ala; p.Val283Ala	39.5%	14%		NBS
PID4	*VLCAD*	c.848 T>C; c.1444_1448delAAGGA; c.1511_1516delAGAGG	p.Val283Ala; p.Lys482AlafsX78; p.Glu504_Ala505del	36.5%	12%		NBS
PID5	*VLCAD*	c.1159G>T; c.1269 + 1G>A	p.Va1387Phe; p.?^c^	106.5%	17%		Sibling screening
PID6	*VLCAD*	c.266delC; c.848 T>C	p.Pro89HisfsX228; p.Val283Ala	29.0%	< 7%		NBS
PID7	*VLCAD*	c.779C>T; c.848 T>C	p.Thr260Met; c.848 T>C	36.5%	< 7%		NBS
PID8	*VLCAD*	c.1411 T>C; c.1411 T>C	p.Phe471Leu; p.Phe471Leu	—	18%		NBS

CPT2D	Gene	Nucleotide change	Protein change		Enzyme activity CPT2^b^		Diagnosis
PID9	*CPT2*	c.200G>C; c.680C>T	p.Ala67Gly; p.Pro227Ley	33.5%	21%		Diagnosed symptomatically

LCHADD	Gene	Nucleotide change	Protein change		Enzyme activity^b^		Diagnosis
					LCHAD	LCKAT	
PID10	*HADHA*	c.982G>A; c.1072C>A; c.1528G>C	p.Cys328Arg; p.Gln358Lys; p.Glu510Gln	82.0%	26%	88%	Missed by NBS, diagnosed symptomatically
PID11	*HADHA*	c.1528G>C; c.1528G>C	p.Glu510Gln; p.510Gln	28.0%	42%	89%	NBS
PID12	*HADHA*	c.1528G>C; c.1528G>C	p.Glu510Gln; p.510Gln	24.0%	23%	161%	NBS
PID13	*HADHA*	c.1528G>C; c.2099.delG	p.Glu510Gln; p.Gly700GlufsX30	—	25%	89%	NBS

LCKATD	Gene	Nucleotide change	Protein change		Enzyme activity^b^		Diagnosis
					LCHAD	LCKAT	
PID14	*HADHB*	c.154A>T; c.1289 T>C	p.(Arg52Trp); p.(Phe430Ser)	106.0%	38%	9%	Diagnosed symptomatically

Abbreviation: lcFAO flux, long‐chain fatty acid β‐oxidation flux.

^a^ lcFAO flux was measured in fibroblasts as an average of 2 replicates and calculated as % of the mean activity in the control fibroblasts analyzed in the same experiment.

^b^ Enzyme activity was measured in lymphocytes, and the mean was expressed as % of the mean of the reference range.

^c^ The variant is positioned at a splice site and has the potential to result in a splicing defect.

### 3.1 Dietary Information

At diagnosis, all patients were advised to adhere to a maximal fasting time according to age and to use additional dietary measures during illness [25]. Patients with LCHADD and LCKATD were additionally treated with a long‐chain triglyceride (LCT)‐restricted (max 10%–15% of total kcals) and medium‐chain triglyceride (MCT)‐supplemented diet (15%–25% of total kcals) leading to 30%–35% of total kcals derived from fat. Most patients with VLCADD and the patient with CPT2D consumed a meal or a carbohydrate‐rich snack within an hour before exercise, but not before routine physical activities such as physical education and cycling to school. Patients with LCHADD and LCKATD had stricter dietary measures with MCT intake prior to sports and sometimes also before routine physical activities. For most patients, intake depended on the activity intensity and length. Before exercise testing, patients consumed a normal pre‐exercise breakfast, including MCT supplementation for patients with LCHADD and LCKATD.

### 3.2 Physical Activity Levels and Muscle Pain in Patients With lcFAOD

Reported participation in an organized sport at least once a week, besides physical education at school, and total weekly exercise time in both patients with lcFAOD and the Dutch population [23, 24] are reported in Table 2. During follow‐up (median: 1.7 years, range: 0–2.4), patients slightly increased their physical activities, with median weekly activity increasing by 20 min after 1 year of follow‐up (*n* = 10) and weekly sporters increasing from 79% (*n* = 14) to 90% after 1 year of follow‐up (*n* = 10). These results indicate that the physical activity levels of patients with lcFAOD were within the range of their healthy peers and increased after recommendations based on exercise testing.

**TABLE 2:** Reported participation in an organized sport at least once a week besides physical education at school and total weekly exercise time in both patients with lcFAOD and the Dutch population.

	Participation organized sport at least once a week (in %)		Total weekly exercise time (in hours, median (range))	
	Age 4–11 years	Age 12–17 years	Age 4–11 years	Age 12–17 years
Dutch population [24, 25]	58%	72%	1.9^a^	4.5^a^
lcFAOD (diagnosed by screening)	100%	50%	4.0 (3–5.5), *n* = 4	3.5 (0–4.9), *n* = 7
lcFAOD (diagnosed symptomatically)	n.a.	100%	n.a.	3.5 (2.5–3.8), *n* = 3

^a^ For the Dutch population, no range was reported [23, 24].

All patients with lcFAOD reported exercise‐induced muscle pain, with 46% of the patients diagnosed by screening and 100% of the symptomatically diagnosed patients reporting at least one episode of debilitating muscle pain. Debilitating muscle pain caused by exercise occurred only (*n* = 5) or mostly (*n* = 3) under specific conditions with clear provoking factors, such as exercising for several hours, consecutive physical activities, unusual exercise, limited food intake, extreme circumstances (e.g., hot or cold temperatures, little sleep) or, retrospectively, a viral infection (Details in Table S1). The first episode of debilitating muscle pain occurred at a median age of 10.9 years for patients with VLCADD diagnosed by screening (range: 9.8–12.0, *n* = 2/8), 7.8 years for patients with LCHADD diagnosed by screening (range 4–10.5, *n* = 3/3), 13.7 years for the patient with LCHADD missed by NBS (*n* = 1/1), 16.7 years for the patient with CPT2D (*n* = 1/1) and 17.1 years for the patient with LCKATD (*n* = 1/1). There was no significant difference in total weekly exercise duration between patients with and without debilitating muscle pain (median of 225 vs. 210 min, *p* = 0.76).

Most patients with lcFAOD (64%) experienced increased CK levels (≥ 1000 U/L) either after exercise or without any apparent cause such as febrile illness during follow‐up (median age of first increased CK measurement: 11.3 years in patients diagnosed by screening (range: 7.7–15.6) and 15.5 years in patients diagnosed symptomatically (range: 13.8–17.1)).

To put these myopathic symptoms in perspective, the frequency and intensity of exercise‐induced muscle pain were recorded in a similar number of children without lcFAOD. These children were healthy (*n* = 6) or were diagnosed by NBS with diseases that are not expected to cause debilitating myopathic symptoms (including MCADD (*n* = 5) and PKU (*n* = 3)). The median age of all children was 14 years (range: 8–17 years). Nine out of 14 (64%) of these children reported exercise‐induced muscle pain (healthy: 50%, MCADD: 80%, PKU: 67%) compared to 100% of patients with lcFAOD. With a median pain score of 4 (on a scale of 0 to 10, range: 2–5), this muscle pain was interpreted as not debilitating in all control children, while debilitating pain was reported in 57% of lcFAOD patients. Concordantly, MCADD, PKU, and healthy children reported being able to continue daily physical activities, albeit sometimes hindered by the muscle pain during exercise, including sports, walking, or biking (*n* = 6, questionnaire option 2 or 3, Supporting Information). One patient reported the influence of muscle pain on going to school but was only mildly hindered during sports, walking/biking, and playing. Therefore, this was not interpreted as debilitating muscle pain.

### 3.3 Maximal Exercise Capacity

Maximal effort during CPET was defined as HR_peak_ > 180 bpm or RER_peak_ > 1.0 [20]. Based on this definition, we excluded the peak values of one CPET from analyses based on a HR_peak_ of 135 bpm, RER_peak_ of 0.99, and post‐exercise lactate of 2.6 mmol/L: the effort was considered submaximal and insufficient for proper interpretation. Four patients reached the RER_peak_ without reaching the HR_peak_. We hypothesized that in patients with lcFAOD, maximal metabolic effort was reached before a maximal heart rate could be attained, and therefore we considered these tests as maximal despite not reaching HR_peak_ during the test. Correspondingly, there were only minor differences in relative WR_peak_ and VO_2peak_ between patients who did or did not reach a HR_peak_ over 180 bpm (Figure S1).

**FIGURE 2:**
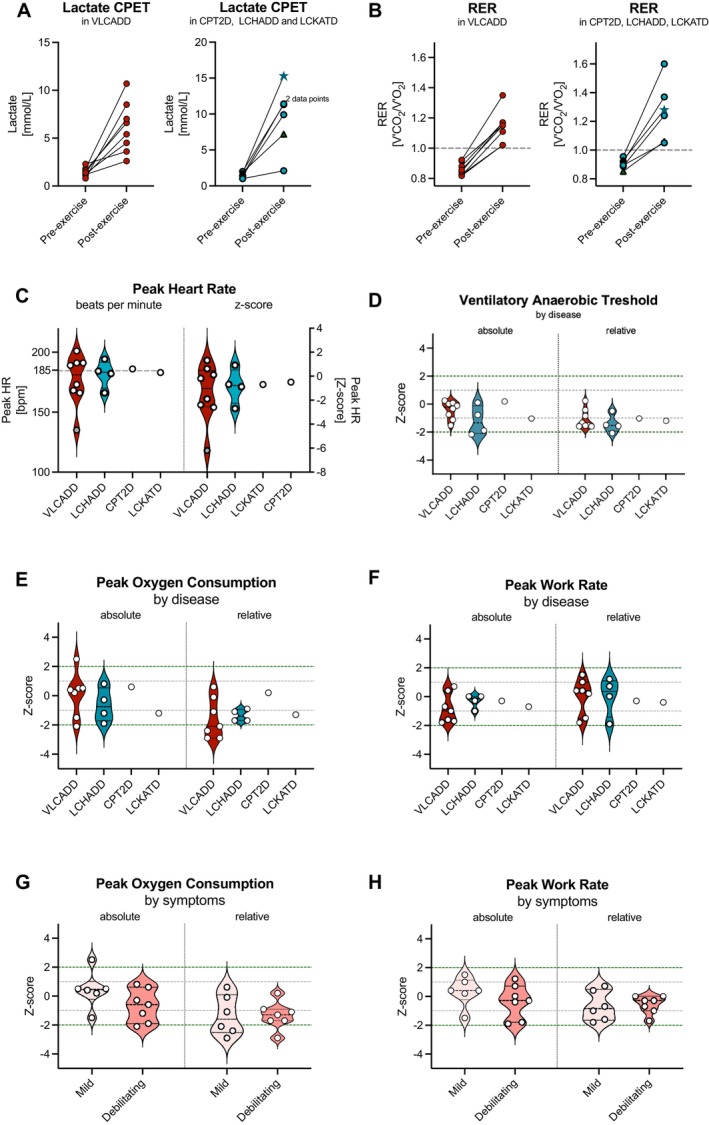
Cardiopulmonary exercise test results. (A) Lactate and (B) respiratory exchange ratios (RER) before and after cardiopulmonary exercise testing (CPET). Red circles: Patients with VLCADD, patients with LCHADD, green triangle: Patient with CPT2D blue star: Patient with LCKATD. (C) Maximal attained heart rate during CPET in beats per minute (bpm, left) and z‐score compared to age and sex matched control data (left). The patient with submaximal effort who was excluded from statistical analyses of peak values is shown as a grey symbol. (D) Absolute and relative (corrected for body mass) ventilatory anaerobic threshold per disorder (VLCADD (red), LCHADD (blue), CPT2D and LCKATD) shown as z‐score compared to age and sex matched control data. (E) Absolute and relative (corrected for body mass) peak oxygen uptake and (F) dynamic muscle strength per disorder (VLCADD (red), LCHADD (blue), CPT2D and LCKATD) shown as z‐score compared to age and sex matched control data. (G) Absolute and relative peak oxygen uptake and (H) dynamic muscle strength in patients with mild muscle symptoms (light pink) compared to patients with at least one episode of debilitating muscle symptoms (dark pink) shown as z‐score compared to age and sex matched control data.

The body mass‐corrected VAT was significantly lower in patients with lcFAOD compared to control data (z‐score − 1.0 (0.8) [median (IQR)], *p* = 0.001, Figure 2D). Average WR_peak_ and VO_2peak_ were similar to control data (WR_peak_: z‐score 0.2 (1.8), *p* = 0.97; VO_2peak_: z‐score 0.2 (1.9), *p* = 0.73). After correction for body mass, median values of both W_peak_ and VO_2peak_ had a z‐score above −2, but were significantly lower in comparison to control data (WR_peak/kg_: z‐score − 0.7 (1.3), *p* = 0.03; VO_2peak/kg_: z‐score − 1.3 (2.8), *p* = 0.005, Figure 2E,F). Between patients with and without debilitating muscle pain, there were no significant differences in median WR_peak_ and VO_2peak_, both for absolute (*p* = 0.30 and 0.30, respectively) and relative values (*p* = 0.59 and *p* = 1.0, respectively, Figure 2G,H). Three out of four patients with LCHADD and the patient with LCKATD showed higher post‐exercise lactate levels than patients with VLCADD and CPT2D (Figure 2A), while WR_peak_ was similar (Figure 2F, Table 3).

**TABLE 3:** Cardiopulmonary exercise test results in patients with lcFAOD.

PID	Post‐exercise lactate^a^	PeakHR	RERpeak	Watt/kg peak	Wattpeak	VO_2_peak/kg	VO_2_peak	VAT
	(mmol/L)	bpm (z‐score)		Watt/kg (z‐score)	Watt (z‐score)	ml/kg/min (z‐score)	L/min (z‐score)	% of pred. VO_2_peak
PID1	5.4	173 bpm (−1.9)	1.11	2.7 (−1.0)	184 (1.0)	29.5 (−2.1)	2.1 (0.5)	43.8
PID2	4.5	168 bpm (−2.4)	1.13	2.7 (−1.7)	130 (−1.8)	31.6 (−2.9)	1.6 (−2.1)	39.4
PID3	10.7	191 bpm (0.1)	1.30	3.4 (−0.7)	200 (0.2)	42.2 (−1.1)	2.6 (0.5)	47.1
PID4	3.6	166 bpm (−2.6)	1.00	3.3 (0.4)	122 (0.4)	39.9 (−0.1)	1.5 (0.2)	49.4
PID5	2.6	135 bpm (−6.2)	0.99	3.1 (−1.3)	174 (−1.2)	30.6 (−3.0)	2.1 (−1.5)	46.6
PID6	8.5	189 bpm (−0.2)	1.16	2.9 (−1.8)	250 (0.4)	34.1 (−2.4)	3.1 (0.4)	57.1
PID7	6.6	191 bpm (0.6)	1.07	3.9 (0.7)	158 (1.5)	51.2 (0.6)	2.2 (2.5)	43.5
PID8	7.0	201 bpm (1.3)	1.14	2.7 (−1.6)	124 (−1.5)	31.4 (−2.9)	1.7 (−1.5)	43.5
PID9	7.2	186 bpm (−0.5)	1.03	3.3 (−0.3)	180 (−0.3)	43.3 (0.2)	2.6 (0.6)	29.5
PID10	11.3	184 bpm (−0.7)	1.23	3.9 (0.0)	204 (0.7)	43.7 (−0.9)	2.5 (0.8)	41.6
PID11	9.9	182 bpm (−0.9)	1.21	3.6 (−0.3)	160 (0.0)	38.3 (−1.7)	1.7 (−1.2)	48.4
PID12	11.4	194 bpm (0.9)	1.36	3.6 (0.0)	160 (1.2)	37.8 (−1.7)	1.7 (−0.3)	35.2
PID13	2.1	166 bpm (−2.7)	1.03	2.7 (−1.0)	79 (−1.9)	34.9 (−1.1)	1.1 (−1.9)	49.7
PID14	15.3	183 bpm (−0.7)	1.26	3.6 (−0.7)	244 (−0.4)	40.5 (−1.3)	2.8 (−0.6)	39.9

Abbreviations: bpm: beats per minute, RER: respiratory exchange ratio, WR: work rate in Watt, VO_2_: oxygen uptake, VAT: ventilatory anaerobic threshold, Z‐scores are based on age‐ and sex‐matched control data.

^a^ Post exercise lactate was measured 2 min after termination of the CPET.

### 3.4 Prolonged Endurance Exercise in Patients With lcFAOD

**FIGURE 3:**
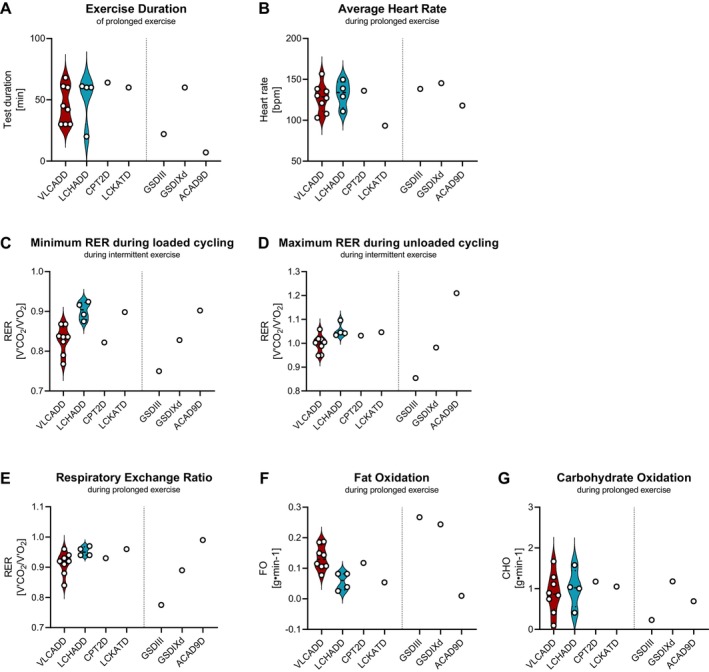
Substrate oxidation during intermittent‐ and prolonged endurance exercise testing. (A) Minimum respiratory exchange ratios (RER) values during loaded cycling during the IXT, (B) maximum RER values during unloaded cycling during IXT, (C) duration of PXT (in minutes), (D) mean heart rate during PXT (in beats per minute (bpm)), (E) mean RER during PXT, (F) mean carbohydrate oxidation during PXT, and (G) mean fat oxidation during PXT in patients with lcFAOD (VLCADD, LCHADD, CPT2D and LCKATD), two patients with GSD (III and IVd), and one patient with ACAD9D.

Eight out of 14 patients with lcFAOD (57%) completed the PXT. The remaining 6 patients stopped after 20 to 45 min of endurance exercise (Figure 3A) because of exhaustion or muscle pain. For one patient with LCHADD (PID 13), the muscle pain during the PXT was similar to her daily‐life pain during cycling or walking.

**FIGURE 4:**
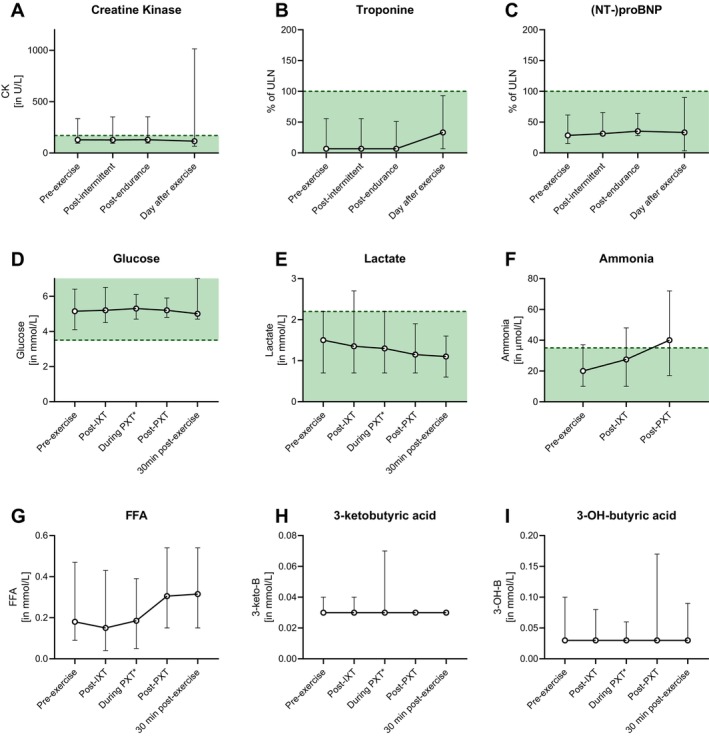
Plasma metabolites during and after intermittent‐ and prolonged endurance exercise in patients with lcFAOD. Creatine kinase (CK) levels (in U/L), (B) troponin levels (in percentage of the upper limit of normal (ULN)) and (C) (NT‐)proBNP levels (in percentage of the ULN) were measured before, during, 30 min, and ~ 24 h after exercise testing. (D) Glucose (in mmol/L), (E) lactate (in mmol/L), ammonia (in mmol/L), (F) free fatty acids (FFA, in mmol/L), (H) 3‐ketobutyric acid (in μmol/L) and I) 3‐hydroxybutyric acid (in μmol/L) were measured before, during, and 30 min after exercise testing. Normal ranges are shown in green.* Measurements during PXT were collected at T = 50, only in patients exercising over 50 min in total (*n* = 10).

The protocol was designed to keep subjects below the anaerobic threshold and maximize lipid consumption as fuel for the exercising muscles. With a median HR of 68% of the predicted maximal HR (range: 49%–83%, Figure 3B for average HR in bpm) and 72% of the attained maximal HR during CPET (range: 51%–81%), the expected optimum for fat oxidation during exercise was approximately reached (61.0% and 66.8% for adolescent boys and girls, respectively [26]). Fat oxidation was further evidenced by a rise in free fatty acids (FFA) in almost all patients (13/14), whereas ketone production was minimal, due to the lcFAOD (Figure 4H,I). As expected, disease‐specific acylcarnitine levels rose concomitantly during exercise in all but two patients with lcFAOD (Figure S2).

In the 30 min after exercise, acylcarnitine concentration further increased in 3 out of 4 patients with LCHADD and in the patient with LCKATD, whereas acylcarnitines remained stable or decreased in the other patients (Figure S2).

None of the patients experienced hypoglycemia or significantly increased CK or cardiac enzyme concentrations during (*n* = 14) or one day after exercise (*n* = 12) (Figure 4A–D), except a mildly increased CK (1.014 U/L, ref. ≤ 145) 24 h after exercise in the patient with CPT2D, with a decrease to 634 U/L at 48 h. This may relate to an additional field hockey training in the evening after the exercise test. For the two patients with slightly increased CK levels during exercise (up to 352 U/L), CK levels at 24 h after exercise were not available due to unfortunate omission to collect blood (*n* = 1) or sample loss (*n* = 1). None of the patients (*n* = 14) had debilitating muscle pain the day after exercise testing.

### 3.5 Substrate Metabolism

RER values represent substrate metabolism in the entire body, with higher values (1 or higher) corresponding to carbohydrate use and lower values (0.7) to fat oxidation. RER is influenced by exercise duration and intensity, training status, sex, and dietary intake, amongst others [27].

In our study, RER values were significantly higher in patients with LCHADD and LCKATD than in patients with VLCADD and CPT2D. Median RER_nadir_ values during loaded cycling in the IXT were 0.90 in patients with LCHADD and LCKAT and 0.84 in patients with VLCADD and CPTD (*p* = 0.004); median RER_peak_ values during unloaded cycling in the IXT were 1.05 and 0.93, respectively (*p* < 0.001) and mean RER values during prolonged endurance exercise were 0.96 and 0.92, respectively (*p* = 0.012, Figure 3C–E). Correspondingly, patients with LCHADD and LCKATD showed a significantly lower median fat oxidation compared to patients with VLCADD and CPT2D (0.05 g·min^−1^ vs. 0.12 g min^−1^, respectively, *p* = 0.004) and higher, albeit not significantly, median carbohydrate oxidation (1.04 vs. 0.90 g min^−1^, respectively, *p* = 1.0) during PXT (Figure 3F,G).

Neither RER nor fat oxidation during PXT correlated with lcFAO‐flux, a measure of residual β‐oxidation (not shown). During PXT, lactate levels remained low, indicating no or minimal contribution of anaerobic oxidation (Figure 4E). Ammonia only mildly increased, putatively through AMP deamination during exercise (Figure 4F) [28].

To validate our substrate oxidation results, we compared our results to patients with metabolic disorders affecting other metabolic pathways: the mitochondrial disease ACAD9D and diseases in carbohydrate metabolism GSD types III and IXd. Compared to our patients with lcFAOD, the patient with ACAD9D had higher RER_nadir_ during loaded phases (0.90) and RER_peak_ values during unloaded phases (1.21) in the IXT (Figure 3C,D). During PXT, which was preliminarily terminated after only 4 min, fat and carbohydrate oxidation were both reduced (0.01 g min^−1^ and 0.69 g min^−1^ respectively), reflecting mitochondrial dysfunction, with upregulated cytosolic glycolysis, indicated by the high mean RER of 0.99 and lactate production during prolonged exercise (up to 7.7 mmol/L, Figure 3E,F,G).

In contrast, patients with GSD‐III and GSD‐IXd had lower RER_nadir_ values during the loaded phases in the IXT compared to patients with lcFAOD (0.75 and 0.83, respectively, Figure 3C). Concurrently, fat oxidation during PXT was remarkably higher: 0.27 and 0.24 g min^−1^, respectively, putatively compensating for the impaired glucose availability from glycogen (Figure 3F). The more severely affected patient with GSD‐III had a low RER_peak_ value of 0.85, a low RER_mean_ of 0.78, and low carbohydrate oxidation (0.23 g min^−1^, Figure 3D,E,G), concurring with the impaired glycogen metabolism. The milder patient with muscle‐specific GSD IXd had higher RER_peak_ values during the unloaded phases in the IXT of 0.98, RER_mean_ of 0.89, and carbohydrate oxidation of 1.18 g min^−1^ during prolonged exercise. This may indicate that muscle metabolism only partly contributes to these whole‐body measurements.

## 4 Discussion

In this study, we demonstrate that despite early diagnosis by screening and subsequent initiation of dietary measures, patients with lcFAOD remain at risk to develop debilitating exercise‐induced muscle pain. Evaluation of our national adolescent cohort of patients with lcFAOD revealed that exercise‐induced symptoms primarily occurred when there was an additional factor increasing metabolic demand or reducing alternative substrate availability (e.g., infection, extreme temperatures, insufficient sleep, engaging in new/intense types of exercise, inadequate intake before/during exercise). Interestingly, awareness of the metabolic condition and experiencing exercise‐induced muscle pain did not substantially influence the exercise patterns of adolescent patients with lcFAOD either diagnosed by screening or symptomatically compared to the general Dutch population. Exercise capacity during CPET was highly variable, with some patients exhibiting a decreased capacity.

In our cohort of adolescent patients with lcFAOD, predominantly diagnosed by screening (79%), all reported exercise‐induced muscle pain, which patients often mentioned as “normal muscle soreness.” For comparison, we conducted a small exploratory questionnaire study (*n* = 14) and found that 64% of healthy children and NBS‐diagnosed MCADD/PKU patients also reported exercise‐induced muscle pain. However, almost half of the patients with lcFAOD diagnosed by screening (46%), particularly those with LCHADD (100%), and all patients with symptomatically diagnosed lcFAOD had experienced at least one episode of muscle pain severe enough to impair daily activities or necessitate hospitalization, sometimes with documented rhabdomyolysis. Albeit potentially influenced by a selective memory as a result of the retrospective questionnaire, in healthy children and in patients with MCADD/PKU, muscle pain did not impair daily activities, but mainly hindered subsequent exercise.

In the patients who reported debilitating muscle pain, neither CPET nor PXT provoked severe muscle pain or significantly elevated CK levels the day after exercise. This may relate to preliminary termination of the PXT in our patient cohort (57%). Preliminary termination of the test may be a consequence of the patients' young ages, their motivation and perseverance, or a (subconscious) prevention to develop (debilitating) muscle pain. However, others have reported changes in CK levels in patients with lcFAOD 24 h after performing a PXT of in total 40 min [11]. Of these patients, 43% stopped the PXT preliminary. These results indicate that this test can stress patients with lcFAOD sufficiently to explore the borders of safe exercise. This may relate to the inclusion of mostly relatively mildly affected patients in our cohort.

Altogether, the conducted exercise during the tests was within safe limits under our controlled well‐fed conditions without additional provoking factors. When reduced capacities are observed, this can be used as a starting point for physical training recommendations. Considering individual patients' sports preferences will enhance adherence and joy in the physical training recommendations. Patients should, however, exercise caution when additional provoking factors are present. It is important to incorporate this consideration into exercise recommendations, alongside dietary recommendations.

Despite symptoms, most patients with lcFAOD maintained a level of weekly exercise similar to their healthy peers. Maximal exercise capacity, however, was highly variable between patients, with slightly decreased relative exercise capacities. These results align with reports of impaired exercise capacity of varying severity in symptomatic adult patients with lcFAOD [10, 29, 30, 31, 32, 33]. A confounding factor in population exercise studies is the fitness of the participants. To increase insight into this confounding factor, we included patients with other metabolic diseases and compared reported weekly activity levels to the Dutch population. We found that patients were able to increase their weekly activity levels with specific guidance in the first year after exercise testing, but continued monitoring of symptoms and exercise capacity of the screening cohort is essential to reveal whether exercise patterns and capacity remain (in the lower range of) normal thanks to the milder nature of NBS diagnosed disease or to early treatment initiation and awareness, or whether they deteriorate as the disease progresses. The wide variability in exercise capacity measured by CPET further highlights the importance of conducting longitudinal follow‐up and exploring therapeutic (training) options for individual patients rather than relying on group‐level testing.

The PXT studies revealed signs of impaired fat oxidation and increased carbohydrate oxidation in patients with lcFAOD compared to patients with other metabolic disorders. Despite the potential influence of varying PXT duration and dietary measures, these results concurred with the known metabolic defects and with previously reported findings [7]. Surprisingly, there were consistent indications of more strongly reduced fat oxidation and increased carbohydrate usage in patients with LCHADD and LCKATD, using LCT‐restricted and MCT supplemented diet, and MCT‐supplementation prior to exercise, compared to patients with VLCADD and CPT2D (e.g., higher post‐CPET lactate concentrations, higher minimum and peak RER values during IXT, and higher carbohydrate and lower fat oxidation and RER during PXT). Combined with the finding that all patients with LCHADD experienced debilitating muscle symptoms at earlier ages than patients with VLCADD, these results suggest differences in myopathic phenotype and substrate oxidation between the diseases, which deserve further investigation. There may also be a role for the individual patients' dietary measures. Although MCT supplementation before or during exercise has previously been reported to decrease RER in patients with lcFAOD [12] and slow carbohydrate oxidation in healthy subjects [34], an LCT restricted diet and increased carbohydrate intake may hypothetically increase RER and carbohydrate oxidation. A standardized diet before exercise testing would reduce dietary influences and is required to further validate potential differences in substrate oxidation.

The RER values represent substrate metabolism in the entire body. However, to further improve our understanding of peripheral oxygen extraction and utilization, the development of reliable and valid methods to accurately assess cardiac output or arteriovenous oxygen difference, for example, as reported by Taivassalo et al. [35], is needed. Future studies using these methods would increase valuable insights in peripheral muscular mitochondrial dysfunction not only in metabolic conditions but also in other (neuro)muscular disorders.

While CPET and PXT are well‐established methods for exercise testing, results are highly dependent on patient participation and disease severity. In our study, only eight adolescents completed the PXT. While premature termination of the PXT may be partly due to the disease, the reported normal weekly quantity of physical exercise at home and the absence of laboratory abnormalities suggest that patient participation may also play a role. This often preliminary termination makes the PXT less suitable for long‐term monitoring of disease progression or therapeutic effectiveness. To address these challenges, we added intermittent exercise with a predetermined workload to our PXT protocol. We hypothesized that this approach is more feasible for patients with a severe clinical phenotype and less dependent on individual patient participation. Moreover, intermittent exercise might better reflect daily‐life activities, especially during childhood [36]. With this, IXT can improve objective and quantitative longitudinal follow‐up and potentially serve as a valuable tool for future therapy testing. Inclusion of more severe patients and healthy controls and repetitive longitudinal testing will help determine the utility of IXT for long‐term monitoring patients with lcFAOD.

In summary, adolescent patients with lcFAOD reported normal home exercise patterns, despite debilitating exercise‐induced muscle symptoms and even rhabdomyolysis, which especially occurred when additional provoking factors were present. Encouraging physical activity within individual limits may help to prevent a decline in exercise capacity and optimize social engagement. However, we advise considering the risk of metabolic derangement under stressful conditions. Further research, involving the inclusion of more patients (including severe cases), and age, gender, and physical activity‐matched healthy children, standardized (pre‐exercise) diet, and individual repetitive testing, is necessary to delineate the natural disease course of NBS‐lcFAOD populations and identify prognostic factors that may guide personalized exercise recommendations.

## Author Contributions

**Marit Schwantje, Marco van Brussel, Tim Takken, Monique G. M. de Sain‐van der Velden, Mirjam Langeveld, Gepke Visser, Sabine A. Fuchs:** conceptualization and methodology. **Marit Schwantje, Sabine A. Fuchs, Marco van Brussel:** collection of data, formal analysis, and investigation. **Marit Schwantje, Sabine A. Fuchs, Marco van Brussel:** writing – original draft. **Marit Schwantje, Marco van Brussel, Tim Takken, Monique G. M. de Sain‐van der Velden, Mirjam Langeveld, Gepke Visser, Sabine A. Fuchs:** writing – review and editing. **Marit Schwantje, Sabine A. Fuchs, Marco van Brussel:** visualization.

## Ethics Statement

The performance of a cardiopulmonary exercise test (CPET) and a combined intermittent‐ and prolonged‐endurance exercise test as part of the clinical follow‐up and the use of clinical data for research were approved by the medical ethics committee of the University Medical Center Utrecht (MEC20‐335C).

## Consent

Written informed consent was obtained from all patients/parents.

## Conflicts of Interest

The authors declare no conflicts of interest.

## Supporting information


Data S1.

## Data Availability

The data that support the findings of this study are available on request from the corresponding author. The data are not publicly available due to privacy or ethical restrictions.
