# Supplementary material for: Myopathic Symptoms and Exercise Tolerance in Adolescent Patients With Long‐Chain Fatty Acid Oxidation Disorders
Source: J Inherit Metab Dis. 2025 Jul 29;48(5):e70070. doi: 10.1002/jimd.70070 (PMC12308107; doi:10.1002/jimd.70070)
Supplement: Supplementary file 1 — Data S1. [file JIMD-48-0-s001.pdf]

Supplementary Materials

Myopathic Symptoms and Exercise Tolerance in Adolescent Patients with Long-Chain Fatty Acid Oxidation Disorders

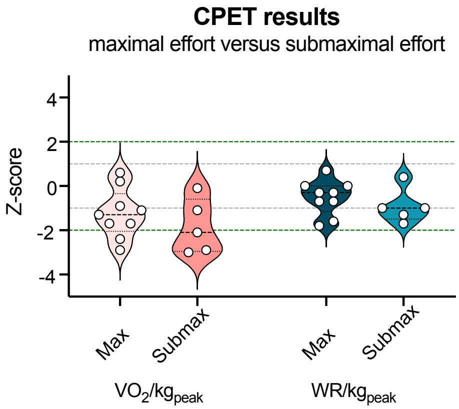

Supplementary Figure 1. Maximal and submaximal effort.

Results of cardiopulmonary exercise tests in patients who reached maximal effort and who did not reach maximal effort, and thus performed a 'submaximal' exercise test, for relative peak oxygen consumption (VO2/kg-peak, pink) and dynamic muscle strength (W/kg-peak , blue). Efforts were considered maximal if HRpeak was > 180 bpm.

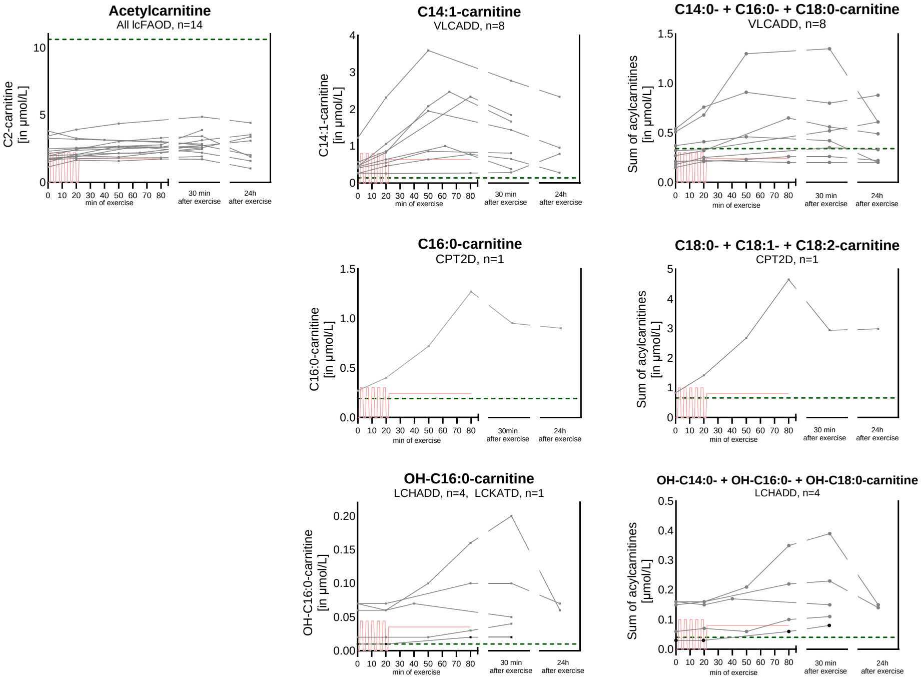

Supplementary Figure 2. Plasma acylcarnitines during and after intermittent- and prolonged exercise in patients with lcFAOD.

A) Acetylcarnitine (C2, in µmol/L) concentrations in all patients with lcFAOD, B) C14:1-carnitine and the sum of C14:0, C16:0 and C18:0-carnitine (in µmol/L) in all patients with VLCADD, C) C16:0-carnitine and the sum of C18:0-, C18:1, and C18:2-carnitine (in µmol/L) in the patient with CPT2D and D) Hydroxy (OH)-C16-carnitine and the sum of OH-C14:0-, OH-C16:0-, and OH-C18:0-carnitine (in µmol/L) in patients with LCHADD (grey) and LCKATD. (black) Acylcarnitine concentrations were measured before, during, 30 minutes after and ~24 hours after exercise testing.

PID	Mild muscle pain	Reported Frequency	Activity	Invalidating muscle pain	Reported Frequency	Potential trigger	Peak CK If >1000 U/L	Sports	Total minutes /week	Follow up (years)	Clinical symptoms during follow-up
PID1	Yes	Several times a year	Physical education	No	n.a.	n.a.	-	No	0	2.3	No muscle symptoms
PID2	Unclear	n.a.	n.a.	Yes	Several times a year	Combination of physical activities, cold weather	2.400	Soccer	292.5	2.1	Invalidating muscle pain<br>CK: 24.000 U/L<br>Trigger: decreased intake, prolonged exercise
PID3	No	n.a.	n.a.	No	n.a.	n.a.	-	No	0	1.7	No muscle symptoms
PID4	Yes	Twice a month	Organized sports (soccer)	No	n.a.	n.a.	-	Soccer, tennis, dancing	330	1.2	No muscle symptoms
PID5	Yes	Unclear	Organized sports (soccer)	Yes	Several times a year	Organized sports (soccer) with decreased intake	1.276	Soccer	210	1.2	Invalidating muscle pain;<br>CK: not measured;<br>Trigger: decreased intake, exercise.
PID6	Yes	Once a month	Organized sports (soccer)	No	n.a.	n.a.	6.790	Soccer, running	280	0.9	No muscle symptoms
PID7	No	n.a.	n.a.	No	n.a.	n.a.	-	Soccer	240	0.7	No muscle symptoms
PID8	No	n.a.	n.a.	No	n.a.	n.a.	-	No	0	0.8	No muscle symptoms
PID9	Yes	Less than once a year	Organized sports for several hours (hockey)	No	n.a.	n.a.	1.033	Hockey	230	2.4	Invalidating muscle pain / rhabdomyolysis;<br>CK: 43.140 U/L;<br>Trigger: decreased intake, viral infection, exercise.
PID10	Yes	Several times a year	Organized sports, (soccer)	No	n.a.	n.a.	-	Fitness at home	210	1.8	Invalidating muscle pain;<br>CK: not measured;<br>Trigger: exercise, first training.

PID	Mild muscle pain	Reported Frequency	Activity	Invalidating muscle pain	Reported Frequency	Potential trigger	Highest CK If > 1000 U/L	Sports	Total minutes /week	Follow up (years)	Clinical symptoms during follow-up
PID11	Yes	Once	Several days of intensive exercise	Yes	Once	Combination of physical activities, cold weather	-	Soccer	210	1.6	Several episodes of invalidating muscle pain;<br>CK: 1.440 U/L;<br>Trigger: trampoline jumping, improvement of symptoms upon increased pre-exercise MCT.
PID12	Yes	Once a week	Organized sports (soccer)	Yes	Once	Combination of physical activities, cold weather	42.900	Soccer	180	1.6	Invalidating muscle pain / rhabdomyolysis;<br>CK: 60.000 U/L;<br>Trigger: little sleep, hot weather, exercise.
PID13	Yes	Unclear	During biking or cycling >30 min	Yes	Several times a year	Prolonged exercise / playing (whole day), hot weather	6.083	Turning	240	1.8	Invalidating muscle pain;<br>CK: not measured;<br>Trigger: exercise.
PID14	Yes	Unclear	Unclear	Yes	Once	Combination of physical activities / playing, with decreased intake, little sleep	157.000	Tennis	150	0	n.a.

Supplementary Table 1. Clinical characteristics of included patients with lcFAOD.
Abbreviations: CK: creatine kinase, U/L: units/liter.

QUESTIONNAIRE OF THE FREQUENCY AND INTENSITY OF EXERCISE-INDUCED MUSCLE SORENESS

Translated to English

My name is ....[Fill in your name]

I am .... years old [Fill in your age]

I am (a) .... [Fill in: Boy/Girl/Non-binary/Other]

Part 1. Muscle pain/soreness

1) Have you ever experienced muscle pain or soreness?

- o Yes
- o No

If you never experience muscle pain or soreness, you can skip the following questions and continue with **question 14 at page 4.**

2) How often do you experience muscle pain without exercising?
(Choose the option that best describes your experience in the past year)

- o Every day
- o A few times per week
- o A few times per month
- o Once a month or less
- o Never

3) How often do you experience muscle pain after exercising?
(Choose the option that best describes your experience in the past year)

- o Every day
- o A few times per week
- o A few times per month
- o Once a month or less
- o Never

4) After which sport or sports do you get muscle pain (especially)?
[write the sports]..
....

5) How long do you need to exercise (approximately) to get muscle pain?
(Fill in what best describes your experience in the past year)

Approximately.......... minutes

6) Do you sometimes need to stop exercising earlier than other children because of muscle pain?
(*Choose the option that best describes your typical month)*

- o Yes always
- o Yes, mostly
- o Yes, sometimes
- o No, never

7) I get muscle pain after exercising...
(Choose the option that best describes your experience in the past year)

- o Always
- o Most of the time
- o Sometimes
- o Never

8) **On a scale from 0 to 10, how severe is your muscle pain when it is at its worst?**
Circle the number or face that best represents your muscle pain in the past year. (0 is no pain, 10 is the worst pain imaginable)

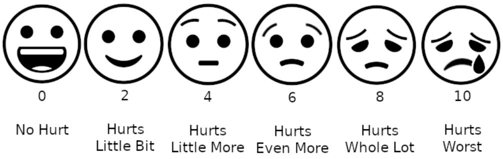

9) **Where in your body do you (especially) experience muscle pain?**
(Write what best describes your experience in the past year)
[write down the areas] ....
....

10) **When does the muscle pain you get from exercising usually start?**
(Choose the option that best describes your experience in the past year)
- o During exercise
- o On the same day, after exercise
- o The next day
- o Other, namely ....[write your description]

11) **How long does it take for the muscle pain to go away?**
(Choose the option that best describes your experience in the past year)
- o Within a few hours
- o After 1 day
- o After 2 days
- o Longer, namely ....[write your description]

12) **Are there things you can no longer do because of the muscle pain when it's at its worst?**
(Check the option that best describes your experience in the past year.)

	I can do this well when I have muscle pain.	I can do this slightly less well because of the muscle pain.	I can do this much less well because of the muscle pain.	I can no longer do this because of the muscle pain.
Sports	☐	☐	☐	☐
Walking or cycling	☐	☐	☐	☐
Being in school all day	☐	☐	☐	☐
Playing and doing fun things	☐	☐	☐	☐

13) Are there special situations in which you get muscle pain faster or more often?
(Check the option that best describes your experience in the past year.)

- o No
- o Yes, namely ....
....[describe the situation]

14) Do you sometimes have to stop exercising earlier than other children because you are tired?
(Choose the option that best describes a typical month)

- o Yes always
- o Yes, mostly
- o Yes, sometimes
- o No, never

15) What sports do you do (almost) every week? (e.g., soccer, hockey, tennis)
(Check the option that best describes your experience in the past year.)

Sport 1: ....

How many times a week do you do this sport?

Fill in: .... [Number of times per week]

How long does this sport take each time?

Fill in: .... [Minutes per training/match]

Sport 2: ....

How many times a week do you do this sport?

Fill in: .... [Number of times per week]

How long does this sport take each time?

Fill in: .... [Minutes per training/match]

Sport 3 ....

How many times a week do you do this sport?

Fill in: .... [Number of times per week]

How long does this sport take each time?

Fill in: .... [Minutes per training/match]

Sport 4: ....

How many times a week do you do this sport?

Fill in: .... [Number of times per week]

How long does this sport take each time?

Fill in: .... [Minutes per training/match]

Part 2. Sports and nutrition

Choose the option that best fits you in the past year:

1) Do you pay attention to what you eat before exercising?
- ○ Yes
- ○ No

2) Do you eat before exercising?
- ○ Yes, always
- ○ Yes, usually
- ○ No, usually not
- ○ No, never

3) What do you usually eat before you exercise?
- ○ A regular meal (breakfast, lunch, or dinner)
- ○ An extra sandwich
- ○ A cookie or bar
- ○ Other, namely ....[write your description]

4) How long before exercising do you usually eat?
- ○ Less than 30 minutes before
- ○ More than 30 minutes before

5) What do you usually drink during exercise?
- ○ Nothing
- ○ Water
- ○ Lemonade or sports drink
- ○ Other, namely ....[write your description]

6) What do you usually eat during exercise?
- ○ Nothing
- ○ Cookie or bar
- ○ Fruit
- ○ A sandwich
- ○ Other, namely ....[write your description]

7) What do you usually eat after you exercise?
- ○ A regular meal (breakfast, lunch, or dinner)
- ○ A dessert (if you exercise after dinner)
- ○ An extra sandwich
- ○ A cookie or bar
- ○ Other, namely ....[write your description]

8) How long after exercise do you usually eat something again?
- ○ Within 30 minutes after exercise
- ○ More than 30 minutes after exercise

Part 3. Physical activity (questions for patients aged 8 to 11 years, based on the Short **QUestionnaire** to ASsess Health-enhancing physical activity (SQUASH))
Think about a typical week in the past few months. Together with your parents, fill in how many days per week you did these activities and how much time you spent on each of those days.

Home-school commute

Walking to and from school	I do this .... days/week [fill in the fitting answer]
	It takes ... minutes/day [fill in the fitting answer]

Cycling to and from school	I cycle on a .... [check the fitting box]<br>0 regular bike<br>0 electric bike
	I do this .... days/week [fill in the fitting answer]
	It takes ... minutes/day [fill in the fitting answer]

Physical activities at school

Physcial education at school	I do this .... days/week [fill in the fitting answer]
	It takes ... minutes/day [fill in the fitting answer]

Swimming at school	I do this .... days/week [fill in the fitting answer]
	It takes ... minutes/day [fill in the fitting answer]

*Playing outdoors at school**	I do this .... days/week [fill in the fitting answer]
** After-school care does not count.*	It takes ... minutes/day [fill in the fitting answer]

Free time – at home

Walking/hiking	I do this .... days/week [fill in the fitting answer]
	It takes ... minutes/day [fill in the fitting answer]

Cycling	I do this .... days/week [fill in the fitting answer]
	It takes ... minutes/day [fill in the fitting answer]

*Playing outdoors **<br>**After-school care counts if it concerns playing outdoors.*	I do this .... days/week [fill in the fitting answer]
	It takes ... minutes/day [fill in the fitting answer]

Swimming lessons	I do this .... days/week [fill in the fitting answer]
	It takes ... minutes/day [fill in the fitting answer]

Part 3. Physical activity (Questions for children aged 12 years and older, based on the Short **QUestionnaire** to ASsess Health-enhancing physical activity (SQUASH))
Think about a typical week in the past few months. Together with your parents, fill in how many days per week you did these activities and how much time you spent on each of those days.

Home-school commute

Walking to and from school	I do this …. days/week [fill in the fitting answer]
	It takes … minutes/day [fill in the fitting answer]

Cycling to and from school	I cycle on a …. [check the fitting box]<br>0 regular bike<br>0 electric bike
	I do this …. days/week [fill in the fitting answer]
	It takes … minutes/day [fill in the fitting answer]

Work
Do you have a job?
- ○ Yes
- ○ No

If yes, what kind of job do you have?
……………………………………………………………………[Fill in the fitting answer]

Light and moderately strenuous work<br>*For example: sitting/standing work with occasional walking, such as desk work or light manual work*	I do this …. days/week [fill in the fitting answer]
	It takes … minutes/day [fill in the fitting answer]

Heavy strenuous work<br>*For example: jobs involving regular lifting of heavy objects or laborious tasks*	I do this …. days/week [fill in the fitting answer]
	It takes … minutes/day [fill in the fitting answer]

Physical education at school	I do this …. days/week [fill in the fitting answer]
	It takes … minutes/day [fill in the fitting answer]

House hold work

Light and moderately strenuous household work<br>*For example: standing work, such as cooking, dishwashing, ironing, vacuuming, and grocery shopping*	I do this …. days/week [fill in the fitting answer]
	It takes … minutes/day [fill in the fitting answer]

Heavy strenuous household work<br>*For example: scrubbing floors, beating carpets, and carrying heavy groceries*	I do this …. days/week [fill in the fitting answer]
	It takes … minutes/day [fill in the fitting answer]

Free time

Walking/hiking	I do this …. days/week [fill in the fitting answer]
	It takes … minutes/day [fill in the fitting answer]

Cycling	I do this …. days/week [fill in the fitting answer]
	It takes … minutes/day [fill in the fitting answer]

Do it yourself (DIY)/ Handywork	I do this …. days/week [fill in the fitting answer]
	It takes … minutes/day [fill in the fitting answer]

Gardening	I do this …. days/week [fill in the fitting answer]
	It takes … minutes/day [fill in the fitting answer]
